# Signet ring cell-like diffuse large B-cell lymphoma involving the breast: a case report

**DOI:** 10.1186/s12905-023-02285-4

**Published:** 2023-03-23

**Authors:** Lu Zhang, Qinqin Min, Jiaxin Bi, Xuewen Yu, Yingying Liang, Mumin Shao

**Affiliations:** Department of Pathology, Shenzhen Traditional Chinese Medicine Hospital, the Fourth Clinical Medical College of Guangzhou University of Chinese Medicine, Shenzhen, China

**Keywords:** Diffuse large B-cell lymphoma, Signet ring cell, Breast, Fluorescence in situ hybridisation, Immunofluorescence, Case report

## Abstract

**Background:**

Diffuse large B-cell lymphoma (DLBCL) with signet ring cell components is extremely rare. Here, we present a case of DLBCL with signet ring cell components involving the breast, which can be easily confused with invasive lobular carcinoma of the breast or metastatic signet ring cell carcinoma of gastrointestinal origin.

**Case presentation:**

A 66-year-old woman presented with a painless mass in her left breast. Enhanced magnetic resonance imaging (MRI) of the breast revealed a 42 × 29 × 28 mm mass in the left breast. Histological examination revealed a diffuse or scattered arrangement of round cells mixed with signet ring-like cells. Immunohistochemically, the neoplastic cells were positive for PAX-5, CD79a, CD20, Bcl-6, and MUM-1 but and negative for cytokeratin, ER, PR, E-cadherin, and P120. The Ki-67 proliferation index was approximately 70%. Fluorescence in situ hybridisation (FISH) demonstrated non-rearrangement of *Bcl-2*, *Bcl-6*, and c-*MYC* genes. Immunohistochemistry and FISH examination confirmed the diagnosis of DLBCL. Subsequently, immunofluorescence showed both IgM and IgG deposits in the signet ring-like lymphocytes. After confirming the diagnosis, the patient received four courses of CHOP (cyclophosphamide, doxorubicin, vincristine, and prednisolone) chemotherapy in a specialist hospital and achieved partial remission; however, she unfortunately died of secondary pneumocystis pneumonia infection 3 months later.

**Conclusion:**

Malignant lymphoma with signet ring cell morphology is quite uncommon, and this variant can be a diagnostic pitfall. We emphasise that pathologists should consider lymphoma in the differential diagnosis of malignant breast tumours.

## Background

Signet ring cell lymphoma (SRCL) is a rare morphologic variant of non-Hodgkin lymphoma (NHL), originally described by Kin et al., and is considered a rare morphologic variant of follicular lymphoma [1]. To date, SRCL has been described in most types of NHL, including both T-cell lymphoma and B-cell lymphoma [2, 3]. Diffuse large B-cell lymphoma (DLBCL) with signet ring cell features is extremely rare. We report a case of DLBCL with signet ring cell-like morphology involving the breast and with multiple metastases throughout the body. To the best of our knowledge, this is the first reported case of systemic DLBCL with signet ring cells involving the breast. Moreover, differentiating this tumour from breast lobular carcinoma or metastatic gastrointestinal carcinoma is important.

## Case presentation

A 66-year-old woman presented to our hospital with a 1-week history of a quasi-circular and painless mass in her left breast. Physical examination of the breast revealed an analogous round nodule in the lateral quadrant of the left breast, approximately 4.0 × 3.0 cm in size. It was mobile and well-circumscribed. She had a surgical history of thyroidectomy that was performed around 10 years prior due to a thyroid nodule. In addition, she had a 13-year history of type 2 diabetes and a 3-year history of thrombocytopenia.

**Fig. 1:**
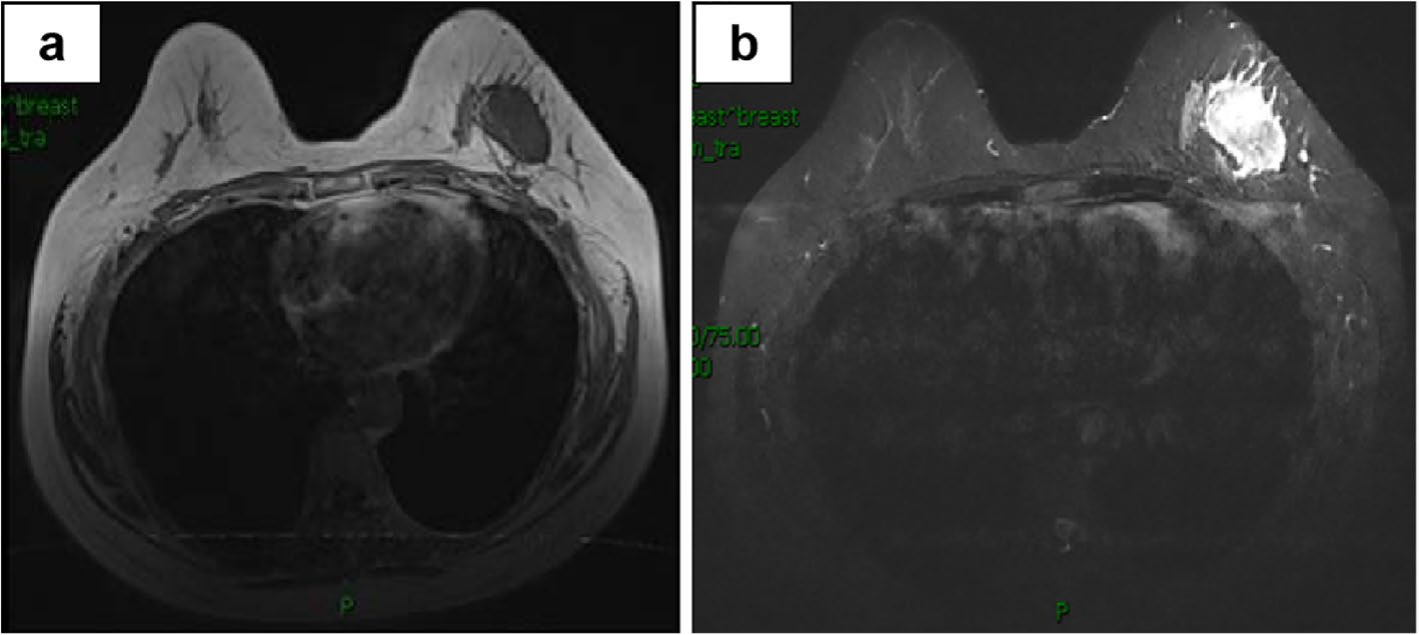
MRI revealed a mass measuring 42 × 29 × 28 mm in the upper outer quadrant of the left breast. **a** On T1 weighted images, the mass was isointense relative to the parenchyma. **b** On T2 weighted images, the mass was slightly hyperintense relative to the parenchyma

Enhanced magnetic resonance imaging (MRI) of the breast revealed a mass measuring 42 × 29 × 28 mm in the upper lateral quadrant of the left breast (Fig. 1a, b). The margin of the lesion was lobulated and ill-defined with respect to the adjacent pectoralis major muscle. There were multiple enlarged lymph nodes in the left axillary region. It was considered to be a breast carcinoma and metastasis could not be excluded. The patient was highly anxious and insisted on surgical treatment. Therefore, radical mastectomy was performed, with intraoperative frozen-section examination.

**Fig. 2:**
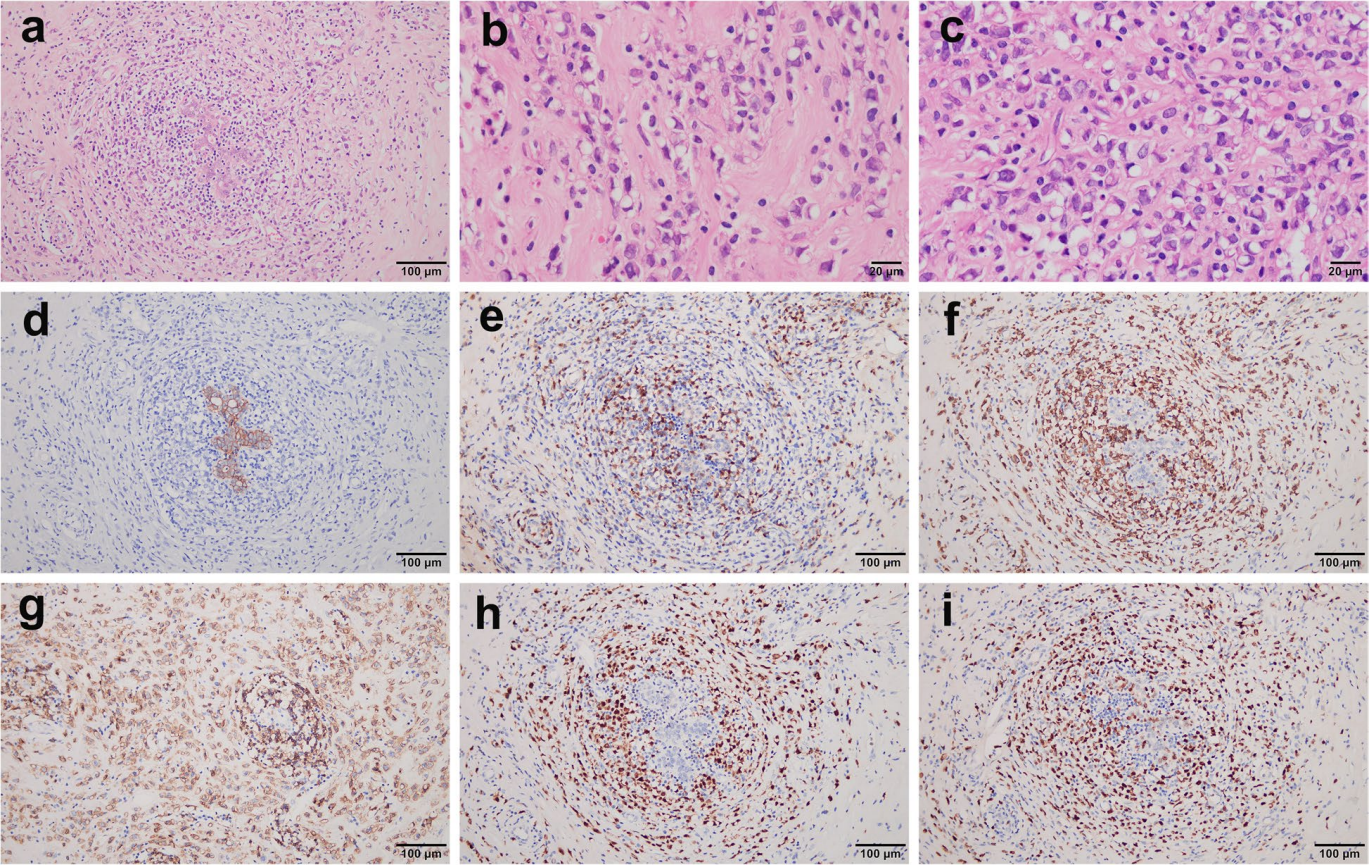
The histology and immunophenotype of breast DLBCL with signet ring cells. **a** The tumour cells are medium to large lymphoid cells with oval to round nuclei, diffusely distributed in the breast interstitium and eroding the breast lobules. **b**-**c** Many of the lymphoid cells have intracytoplasmic vacuoles that press the nucleus, imparting a signet ring appearance. Immunohistochemical stains show that the signet ring cell-like lymphocytes are negative for cytokeratin (**d**) and CD3 (**e**) and positive for CD79a (**f**), CD20 (**g**), and MUM-1 (**H**). The Ki-67 proliferation index was approximately 70% (I)

Macroscopically, the cut surface of the resected breast specimen showed a hoary solid tumour, measuring 3.8 × 1.5 cm, with medium texture and an unclear boundary. Frozen sections from the tumour showed scattered atypical cells accompanied by signet ring-like cells, which are difficult to distinguish from breast lobular carcinoma. Microscopically, the breast lobules were atrophic with small lymphocyte infiltration around the lobules in the paraffin sections. Moreover, lymphoid cells with relatively consistent morphology, medium size, deeply stained nuclei, and less cytoplasm were diffusely distributed in the fibrous stroma and invaded the breast lobules (Fig. 2a). Interestingly, signet ring-like cells were scattered in the mesenchyme (Fig. 2b, c).

**Fig. 3:**
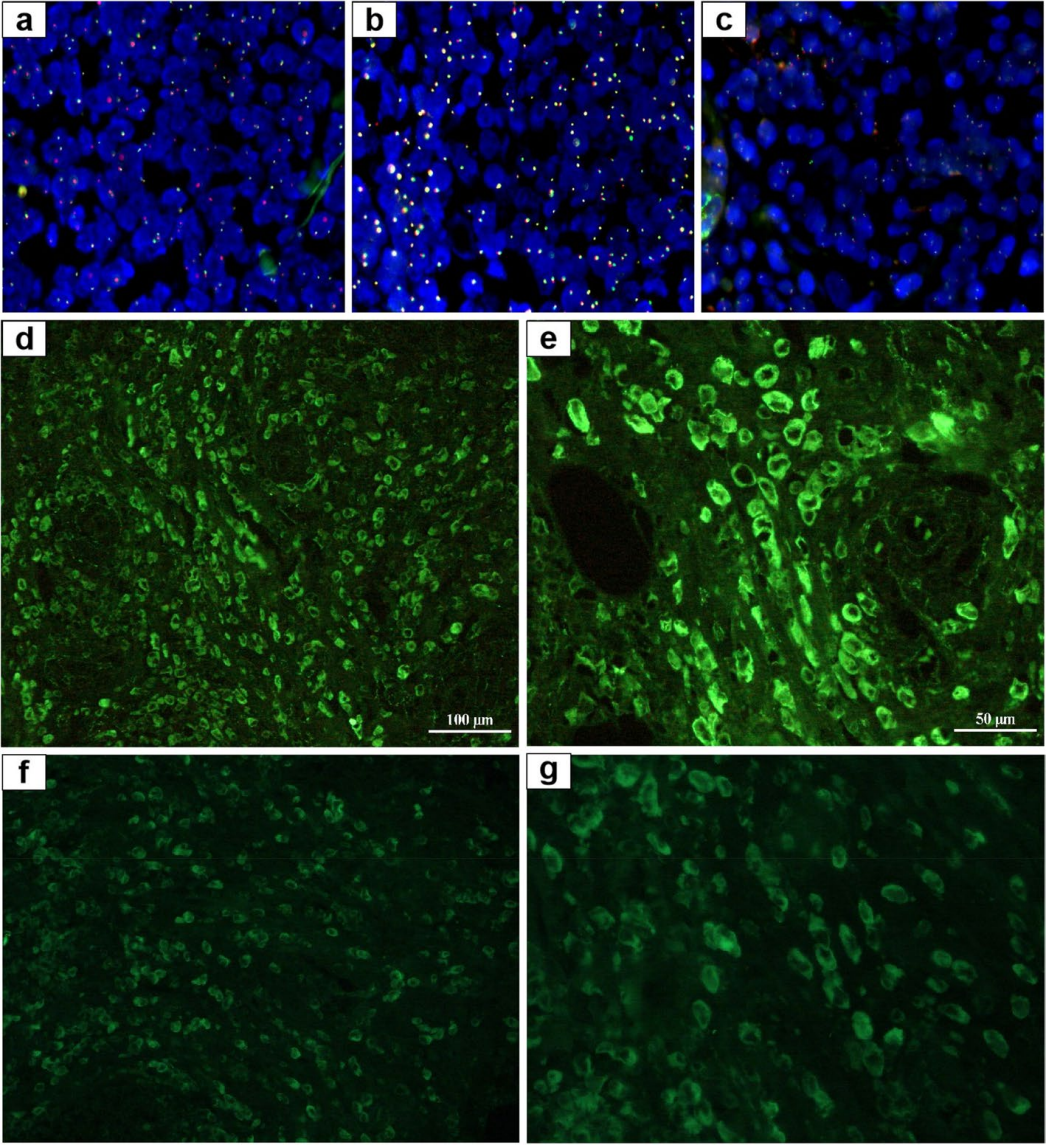
Fluorescence in situ hybridisation (FISH) and immunofluorescence examination of DLBCL with signet ring cells. FISH showing that there is no rearrangement of *Bcl-2* (**a**), *Bcl-6* (**b**), and *c-MYC* (**c**). Immunofluorescence showing both IgM (**d**-**e**) and IgG (**f**-**g**) deposits in the signet ring-like lymphocytes at the same time

Immunohistochemistry (IHC) results showed that the tumour cells did not express cytokeratin (Fig. 2d), ER, PR, E-cadherin, and P120, excluding the diagnosis of invasive lobular carcinoma and metastatic carcinoma. Tumour cells, with no CD3 expression (Fig. 2e), diffusely expressed PAX-5, CD79a (Fig. 2f), and CD20 (Fig. 2g), confirming that they were B-cell-derived, and they expressed CD10, MUM-1 (Fig. 2h), and Bcl-6, suggesting the absence of germinal-centre type B-cells. The proliferation rate was relatively high (Fig. 2i, approximately 70% Ki-67 positive). Fluorescence in situ hybridisation (FISH) demonstrated the absence of *Bcl-2* (Fig. 3a)*, Bcl-6* (Fig. 3b), and *c-MYC* (Fig. 3c) genes, which confirmed the diagnosis of DLBCL. Positron emission tomography-computed tomography (PET-CT) examination revealed the following: (1) Striped hypermetabolic foci in the liver along the left and right bile ducts, (2) nodular hypermetabolic foci on the right scapula, left clavicle, and sternum, and (3) multiple nodular hypermetabolic foci in the hilar area, near the head of the pancreas, and retroperitoneum in the upper abdomen. The results were suggestive of a secondary signet ring cell-like DLBCL of the breast.

To further determine the nature of the vacuoles, immunofluorescence examination was performed. The results revealed that abnormal secretion and accumulation of IgM (Fig. 3d, e) and IgG (Fig. 3f, g) contributed to the prominent signet ring cell morphology.

In addition, interestingly, the non-neoplastic breast tissue surrounding the lymphoma showed various morphological changes. Lymphocyte infiltration was observed around the breast ducts and lobules. Moreover, B-lymphocyte infiltration was seen around the vessels in the interstitium, which was confirmed by immunohistochemical staining for CD20 and CD3. Dense, keloid-like fibrosis and epithelioid myofibroblasts were also seen in the breast interstitium. All these morphological characteristics and a history of type 2 diabetes confirmed a diagnosis of diabetic mastopathy.

After receiving the final diagnosis, the patient went to a specialist hospital for further treatment. She received four courses of CHOP (cyclophosphamide, doxorubicin, vincristine, and prednisolone) chemotherapy and achieved partial remission; however, she unfortunately died of secondary pneumocystis pneumonia infection 3 months later.

## Discussion and conclusions

DLBCL is a high-grade lymphoma and the most common type of NHL [4]. DLBCL is highly aggressive, and patients usually present with rapidly expanding lymph nodes and systemic symptoms that require immediate treatment [4]. Although most patients present with swollen lymph nodes, approximately 40% of DLBCL cases arise from extranodal sites, including gastrointestinal tract, skin, soft tissues, mediastinum, bones, central nervous system (CNS), testes, and breast [5–9]. In addition, advanced DLBCL can involve extranodal tissues or organs, such as the bone marrow, pleura, peritoneum, liver, breast, and CNS, sometimes obscuring the primary site of origin [9, 10].

The spectrum of DLBCL is extensive and includes many morphological variants [10, 11]. Among these variants, DLBCL with signet ring cell characteristics is extremely rare. So far, only about seven cases of DLBCL with signet ring cells have been described in the literature; these originated from the lymph nodes, stomach, breast, orbit, and thigh [12–17]. Our case represents the first reported case of systemic DLBCL involving the breast.

In this case, the patient initially presented with a breast mass. Intraoperative frozen sections revealed aberrant cells arranged in a single row or scattered. Specifically, scattered signet ring-like cells were observed in the mesenchyme. This presented a diagnostic challenge. In fact, when such signet ring-like cells are observed, pathologists are more likely to consider metastatic adenocarcinoma of gastrointestinal origin or breast lobular carcinoma, causing misdiagnosis. The treatment options for these tumours are completely different. Therefore, it is important for pathologists to understand this rare morphological manifestation of DLBCL. When signet ring cell morphology appears, DLBCL should be considered in the differential diagnosis, and IHC studies using lymphoid markers, such as CD3 and CD20, should be performed first. Once the initial IHC suggests lymphoma, more extensive IHC and molecular testing should be performed to further classify the lymphoma. In addition, differentiating between primary and secondary DLBCL of the breast is important. The 2019 version of the World Health Organization diagnostic criteria for primary breast DLBCL includes being confined to one or both breasts, with or without involvement of regional lymph nodes [18]. In this case, PET-CT revealed the presence of metastases in multiple locations. Therefore, the final diagnosis was secondary DLBCL of the breast.

SRCL, most commonly described as a variant of follicular lymphoma, has rarely been described in DLBCL [1, 2]. DLBCL with signet ring cells involving the breast is rare, with only one such case described previously in the literature [13]. Kim et al., who first reported seven SRCL cases, divided SRCL into two types: one type had prominent, clear cytoplasmic vacuoles that contained IgG immunoglobulins, while the other had eosinophilic, hyaline, Russell body-type inclusions composed of IgM immunoglobulins [1]. It is currently believed that signet ring cells in lymphoma are divided into three subtypes: clear vacuole type, Russell body type, and hyaloplasmic deposit type, according to the morphologic and immunohistochemical characteristics [19]. The ultrastructural base of the clear vacuole type is an electron-lucent space limited by a smooth membrane, and deposition of IgG immunoglobulin exists at the periphery of the vacuoles. The Russell body-type is composed of dense granular materials and expanded pools of rough endoplasmic reticulum, related to aberrant secretion and accumulation of IgM. The hyaloplasmic deposit type shows cytoplasmic masses that contain immunoglobulins and correspond to non-membrane-bound hyaloplasmic accumulation of crystalline material. In the present case, immunofluorescence examination demonstrated that both IgM and IgG immunoglobulins were deposited in the cytoplasm of signet ring-like cells, which is inconsistent with previous cases reported in the literature. Our case for the first time demonstrated that signet ring like cells can have multiple types of immunoglobulin deposition at the same time, showing the characteristics of Russell body-type and clear vacuole type, which may represent a unique pathogenesis. To date, no study has reported that abnormal deposition of immunoglobulins has an impact on patient prognosis.

In conclusion, this is a unique case of signet ring cell-like DLBCL involving the breast. This morphological variant can be a serious pitfall for histological diagnosis, and immunohistochemical studies are very useful for differential diagnosis. However, it is important for pathologists to be aware that lymphoma can show a signet ring cell morphology and thus differentiate it from a metastatic signet ring carcinoma or breast lobular carcinoma. All of these can be confused with each other and have different behaviour and management protocols.

## Data Availability

Data sharing is not applicable to this article as no datasets were generated or analysed during the current study.

## Abbreviations

DLBCL: Diffuse large B-cell lymphoma
MRI: Enhanced magnetic resonance imaging
CK: Cytokeratin
ER: Estrogen receptor
PR: Progesterone receptor
FISH: Fluorescence in situ hybridisation
SRCL: Signet ring cell lymphoma
NHL: Non-Hodgkin lymphoma
IHC: Immunohistochemistry
PET-CT: Positron emission tomography-computed tomography
CNS: Central nervous system
